# Application of the pedicle axis view in percutaneous screw placement for type III fracture dislocation of the sacroiliac joint

**DOI:** 10.1186/s12891-023-06333-w

**Published:** 2023-03-31

**Authors:** Zhongzhen Zhao, Guofu Zheng, Xiaodong Chu, Shuofan Wang

**Affiliations:** 1 Hangzhou Normal University, Hangzhou, Zhejiang China; 2 The First People’s Hospital of Linping District, Hangzhou, Zhejiang China

**Keywords:** Pelvic fractures, Sacroiliac joint, Internal fixation, Pedicle axis view

## Abstract

**Aim:**

To investigate the clinical application of axial view projection of the pedicle in percutaneous screw placement for type III fracture dislocation of the sacroiliac joint.

**Methods:**

Percutaneous sacroiliac screw fixation was performed in 29 patients with type III sacroiliac joint fractures under X-ray fluoroscopy (C-arm) using axial view projection of the pedicle after preoperative traction reduction and preoperative preparation. The study included 19 males and 10 females, aged 20 to 75 years old, with a mean age of 42.1 ± 3.4 years.

**Results:**

The total operative time ranged between 44 and 135 min, with a mean of 95.5 ± 9.4 min. The intraoperative fluoroscopy time ranged between 15 and 42 s, with a mean of 25 ± 4.7 s. The intraoperative blood loss ranged between 5 and 10 ml, with a mean of 7.1 ± 1.3 ml. According to the Matta scoring system, excellent outcomes were achieved in 25 cases, whereas good outcomes were achieved in 4 cases. Based on the definition by Neo et al., pedicle screw positions were categorized into four grades: grade 0 (33 screws), grade I (2 screws), grade II (2 screws), and grade III (0 screws). Excellent outcomes were achieved in 94.6% of Grade 0 and I screws. According to Majeed’s functional score, 21 cases achieved excellent outcomes, whereas 8 cases achieved good outcomes. The 29 patients were followed between 3 and 18 months, with a mean of 7.1 ± 1.2 months. All patients achieved anatomical reduction with accurate screw placement and successful healing of their fractures, with no cases of bone penetration or neurovascular injury.

**Conclusion:**

Axial view imaging of the pedicle using fluoroscopy is a convenient and rapid fluoroscopy method for percutaneous screw placement for type III fracture dislocation of the sacroiliac joint, with a high rate of success, good safety, and short fluoroscopy time.

## Introduction

Approximately 3% of total body fractures are pelvic fractures [1], with posterior pelvic rings accounting for 17 – 30% of all pelvic fractures [2]. Accurate reduction and rigid fixation of simple fractures or fracture-dislocations of the posterior pelvic ring facilitate early movement and improve patient lives. Patients treated conservatively may suffer from complications, such as pain and walking impairment. Therefore, surgical treatment is recommended for these patients to reduce the incidence of complications [3], especially in cases of displaced or unstable fractures (or old age) [4]. The currently commonly used surgical methods include open reduction and internal fixation of the pelvic fracture and percutaneous sacroiliac screw placement. Open reduction and internal fixation are rarely used due to greater intraoperative bleeding, severe soft tissue injury, and higher rates of postoperative infection than percutaneous sacroiliac screw placement [4]. Percutaneous sacroiliac screw placement is a minimally invasive method for posterior pelvic ring instability that minimizes intraoperative soft tissue injury, shortens hospital stay and is more cost effective [5]. In light of these advantages, percutaneous sacroiliac screw placement is widely used in clinical practice by orthopaedic surgeons. The upper sacral segment varies significantly among individuals. Cai and Gao [6] classified the upper sacral segment into normal (type I), transitional (type II), and dysplastic (type III) types based on continuous pelvic axial CT. The most common complication during surgery is incorrect placement of sacroiliac screws due to complex changes in the upper sacrum and anatomical variations in the lumbopelvic segment in the dysplastic type (type III). Conventional fluoroscopy is performed in the pelvic inlet, outlet, anteroposterior and lateral views, but these views are cumbersome and obscured by iliac cortical projections in the sacral alar slope. The iliac cortical projection exhibits a significant error rate, and screw misplacement cannot be excluded in the anteroposterior view. Pedicle axial view imaging is simpler to perform than conventional imaging monitoring methods. Moreover, pedicle axial view imaging prevents the influence of the sacral alar slope and ensures the accuracy of fluoroscopy while reducing radiation exposure. Between September 2017 and September 2021, 29 individuals with percutaneous sacroiliac screw fixation were treated with pedicle axial view imaging after posterior pelvic ring reduction, and 37 screws were successfully placed. All treatment outcomes were successful. This study is described in detail below.

## Materials and methods

### General information

In this study, 29 patients with a mean age of 42.1 ± 3.4 years (19 males and 10 females, aged between 20 and 75 years) were recruited. The causes of injury were as follows: accident (14), fall (13), and crush (2). Twenty-four patients had additional fractures at other sites, three had urinary tract injuries, and five had craniocerebral injuries. Eight patients had bilateral fracture-dislocation of the sacroiliac joint. The average time from injury to surgery for all patients was 5.5 ± 0.4 days but ranged from three to seven days. All patients were operated on by a senior surgeon.

### Inclusion criteria

a). Type III sacroiliac joint dislocation according to continuous pelvic axial CT; b) age 18 years or older; c) no sacral nerve injury; and d) no surgical contraindications.

### Exclusion criteria

a). Type I or type II fracture dislocation of the sacroiliac joint according to continuous pelvic axial CT; b). age younger than 18 years old; c). sacral nerve injuries present; d). surgical contraindications.

### Preoperative preparation

All patients were admitted to the hospital for anteroposterior, inlet and outlet pelvic films and plain pelvic radiography and reconstruction. A preoperative examination was performed, and basic vital signs were stabilized. All patients who met the surgical conditions underwent surgery. Based on the preoperative CT, the angle formed by the longitudinal axis of the pedicle and the coronal plane (f angle) and the angle formed by the longitudinal axis of the pedicle and the transverse position (e angle) were measured.All patients cleansing enema and fasting one day before surgery.

### Surgical method

The patient was placed in the prone position on a fluoroscopic table under general anaesthesia. Prior to surgical disinfection, fluoroscopy was performed in the axial position of the pedicle in advance, and the patient position was adjusted to ensure that the operating table did not interfere with the fluoroscopy imaging. If the operating table affected fluoroscopic imaging, we chose the operating table that did not interfere with fluoroscopic imaging.The surgical area was routinely disinfected and draped, followed by direct placement of the sacroiliac screw in patients with proper posterior ring position. For patients with severe and simple fractures or fracture-dislocation of the posterior ring, high-weight traction was conducted for preoperative traction reduction under C-arm machine monitoring before performing sacroiliac screw placement. The fluoroscopic monitoring and screw placement were performed as follows: the tube of the C-arm machine was placed perpendicular to the S1 vertebral body. The C-arm tube was then placed according to the e angle and f angle measured preoperatively such that the S1 pedicle was fluoroscopically ‘oval.‘ The C-arm machine and operating bed were fixed, and a guide wire measuring 3.0 mm in diameter and 18 cm in length was inserted into the ‘oval’ region. The guide wire was directly inserted into the oval region once it became fluoroscopically ‘one point’ and was at the centre of the region. The pelvic outlet and inlet positions were then viewed fluoroscopically after the guide wire penetrated the three layers of the bone cortex to determine its length. Thereafter, an opening was drilled using a hollow drill, and a 7.0 mm hollow screw (Zimmer, USA) was placed in the opening. Fluoroscopic imaging of the pelvic outlet and inlet was repeated to ensure accurate sacroiliac screw placement.

Percutaneous sacroiliac screw fixation was performed on 29 patients. Among them, 21 individuals were treated exclusively with a single sacroiliac joint screw, whereas the remaining 8 cases were treated with two sacroiliac joint screws.

### Postoperative treatment

One day after surgery, the patients received antibiotic prophylaxis to prevent infection. An anticoagulant (low-molecular-weight heparin) was administered on the second day after the surgery. Additionally, pelvic anteroposterior, inlet and outlet examinations and plain pelvic radiography and reconstruction were performed on the second day after surgery. Rehabilitation in bed was started on the same day.

## Results

### Outcome measures and efficacy evaluation criteria

Intraoperative fluoroscopy time, operation time, and intraoperative blood loss were all recorded, and a postoperative CT examination was performed.

**Fig. 1:**
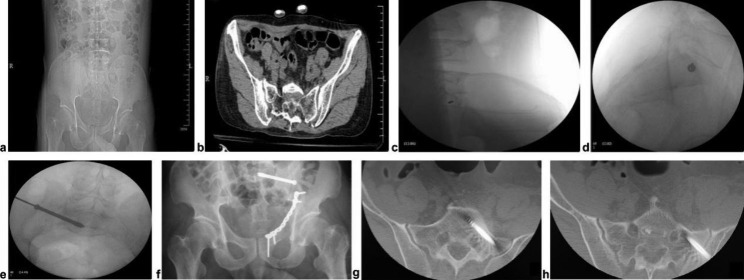
A 43-year-old male with a sacroiliac joint fracture (type III) caused by a fall. Preoperative anteroposterior pelvic radiographs (a) and CT (b) indicate an ipsilateral pubic fracture in a type III sacroiliac joint fracture. Intraoperative X-ray fluoroscopic radiographs (C-arm) during guide wire placement (c), cannulated screw placement (d), and screw insertion (e). Postoperative anteroposterior X-ray (f) and CT radiographs of the pelvis (g, h)

Based on the Matta scoring system, postoperative fracture displacement is classified as excellent if the fracture is less than 4 mm, good if between 4 and 10 mm, fair if between 10 and 20 mm, and poor if more than 20 mm. Patients were clinically evaluated for pain, motor ability, gait, work recovery, and recovery from nerve injury. The patients were also scored using the Majeed functional score, with scores of 85 to 100 classified as excellent, 70 to 84 = as good, 55 to 69 as moderate, and less than 55 as poor. According to the evaluation criteria proposed by Routt, Nork [7], the observed results of the various groups were as follows: Grade 0: the screw was entirely contained within the pedicle; Grade I: the screw penetrated through the pedicle wall by approximately 2 mm; Grade II: the screw penetrated through the pedicle wall more than 2 mm but less than 4 mm; and Grade III: the screw penetrated through the pedicle wall more than 4 mm beyond the pedicle wall. Representative cases are presented in Fig. 1.

### Postoperative conditions

Percutaneous sacroiliac screw fixation was performed in 29 individuals, of which 21 exclusively received single sacroiliac screw placement, whereas the remaining eight cases were fixed with 2 sacroiliac screws. Eight cases were fixed with closed reduction percutaneous cannulated lag screws to the pubic ramus. Eleven individuals underwent open reduction and plate-screw fixation of the pubic ramus, whereas seven individuals underwent percutaneous cannulated lag screw fixation of the pubic ramus and pubic symphysis. Haemorrhagic shock was preoperatively treated in 8 cases. The operative time ranged from 44 to 135 min, with a mean time of 95.5 ± 9.4 min. The intraoperative blood loss ranged from 5 to 10 ml, with a mean of 7.1 ± 1.3 ml. One patient developed a superficial infection of the anterior incision of the pubic symphysis, which healed after dressing changes. No screw breakage occurred within 12 months after surgery. Based on the Matta scoring system, the outcomes of 25 patients were excellent, whereas four were good. The pedicle screw positions were categorized into four grades according to the definition by Griffin, Starr [5], which included grade 0 (33 screws), grade I (2 screws), grade II (2 screws), and grade III (0 screws). Approximately 94.6% of the grade 0 and I screws were scored as excellent. The mean follow-up period for all 29 patients was 7.1 ± 1.2 months but ranged from 3 to 18 months. All patients achieved anatomical reduction with accurate screw placement, no bone penetration, and no neurovascular injury. Overall, all patients underwent full recovery. Based on the Majeed functional score, the treatment outcome was excellent in 21 patients and good in eight patients.

## Discussion

Posterior open reduction and internal fixation with screws are the earliest treatment method for sacroiliac joint fractures, as reported by Letournel [8]. Matta and Saucedo [9] proposed lag screw fixation of the posterior pelvis in 1989. Percutaneous sacroiliac screw fixation is a new type of screw placement technique described recently [10]. However, percutaneous sacroiliac screw fixation is associated with a high risk of injuring the iliac vessels, lumbosacral trunks, and cauda equina in the sacral canal, which occurs in 2–13% of patients who undergo this procedure [11]. The current surgical procedure relies on determining the needle insertion point of the sacroiliac screw using the anteroposterior, inlet, outlet, and lateral views of the pelvis. The standard anteroposterior, inlet, and outlet view of the pelvis is relatively easy to achieve, although the lateral view of the pelvis is prone to penetrate the iliac wing slope during placement of the guide wire, given the iliac cortical density area. Moreover, fluoroscopy was found to be ineffective in a study by Xin, Ma [12]. During anteroposterior and lateral pelvic fluoroscopy, the sacral alar slope is obscured by the sacral ala, making it difficult to determine the position of needle insertion. During percutaneous sacroiliac screw fixation, the L5 nerve around the sacral alar slope is typically the most vulnerable nerve because it is adjacent to the S1 slope and is difficult to move. According to the results of studies by Gardner, Parada [13] and Gardner, Morshed [14], neurovascular injury can be caused by mispositioning of the screw by as little as 4°.

**Fig. 2:**
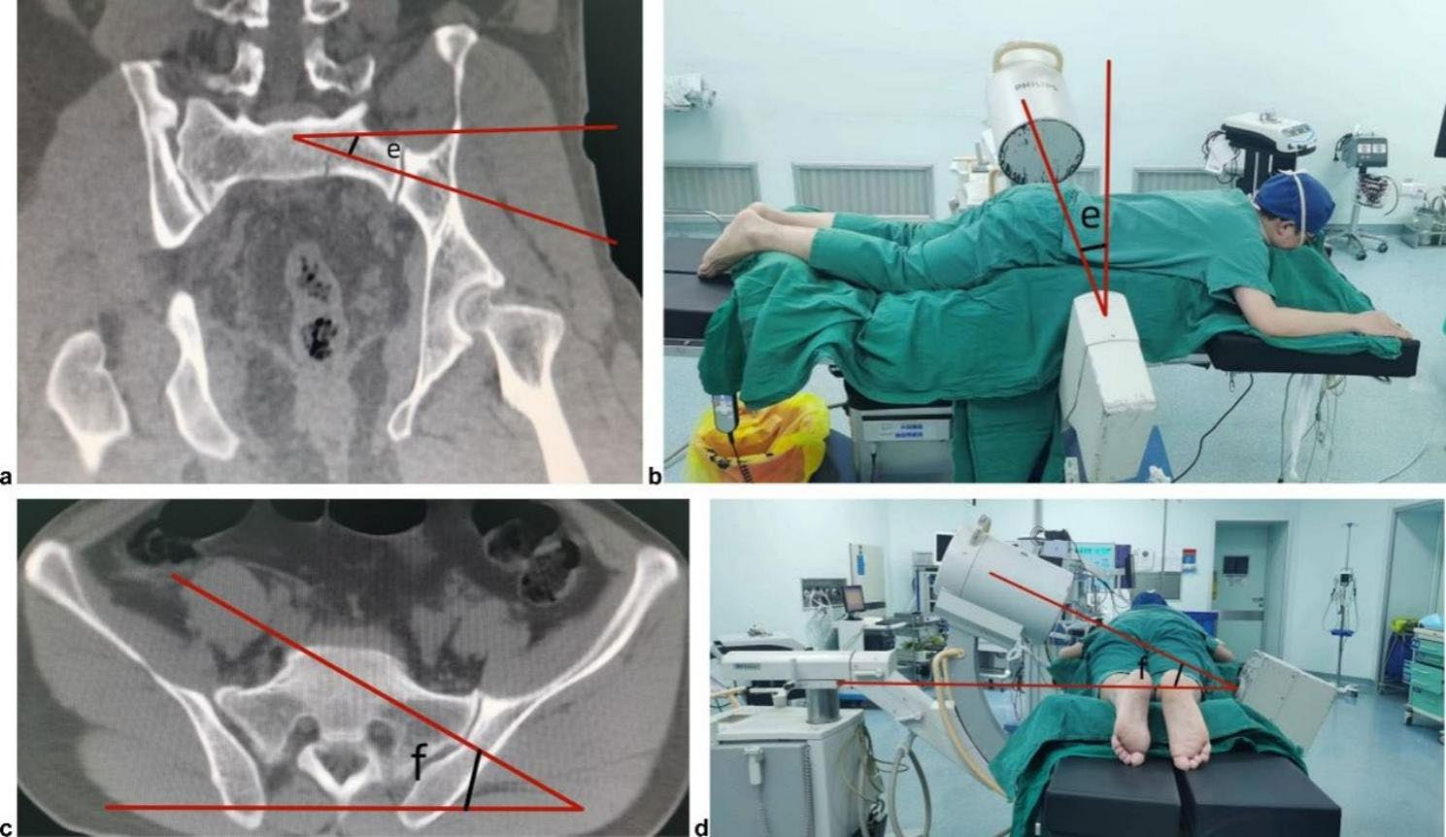
The angle formed by the longitudinal axis of the pedicle and the coronal plane (f angle) and the angle formed by the longitudinal axis of the pedicle and the transverse position (e angle). The tilt angle of the C-arm machine to the head is equal to the e angle, and the horizontal rotation angle of the C-arm machine is equal to the f angle

The risk of neurovascular injury is even higher in type III fracture dislocation of the sacroiliac joint due to the iliac cortical density (ICD) lines of type III sacroiliac joints and the sacral alar slope (SAS). When the SAS is higher than and parallel to the ICD lines, bilateral ICD lines are used as the anterior boundary of the flat screw channel. Conversely, when the SAS is lower than the ICD lines of type III sacroiliac joints, the lowest bilateral SAS lines are used as the anterior boundary of the flat screw channel, and the anterior edge lines of the bilateral sacral nerve root tunnel (SNRT) are used as the posterior boundary of the flat screw channel. The shortest distance between the anterior and posterior boundaries is the width of the flat screw channel. No flat channels (flat channels ≤ 0 mm (zero when the ICD lines were tangential to the anterior edge of SNRT and negative by SNRT)) are observed in type III sacroiliac joints [6]. Current fluoroscopy techniques present particular disadvantages during fluoroscopy of type III sacroiliac joints. The fluoroscopy technique using an axial view of the S1 pedicle significantly overcomes these disadvantages, avoids the effect of the sacral alar slope, reduces its impact on imaging, and reduces the rate of screw placement errors caused by unclear surgical imaging. Intestinal gas has some effect on the anteroposterior and lateral view of the pelvis, but it has little effect on the axial pedicle view. Since the axial pedicle view has less digestive tract tissue in the perspective direction, it has little effect on the axial pedicle view.To obtain clear fluoroscopic images, patients were preoperatively treated with a cleaning enema to reduce intestinal pneumatosis. The preoperative CT fluoroscopy using the p axial view projection of the pedicle determined the angle at which the longitudinal axis of the pedicle and the coronal plane (f angle) were inclined and the angle at which the longitudinal axis of the pedicle and the transverse position (e angle) were inclined. The angles are presented in Fig. 2. During surgery, the patient was positioned in a prone or supine position. Thereafter, the head of the C-arm machine was tilted to an angle equal with the e angle, and the horizontal angle was adjusted to the f angle. Because most patients with type III fractures of the sacroiliac joint cannot be fully positioned in a supine or prone position, the S1 pedicle fluoroscopic radiographs become ‘oval’ after adjusting the angle of the C-arm machine. The C-arm machine was then fixed, and the guide wire was inserted to form a point in the ‘oval’ centre.Bone hammer is superior to electric drill for guidewire placement. A hollow drill was used to make an opening along the direction of the guidewire, and a cannulated screw was placed. A screwdriver was preferred over an electric drill when placing screws. The electric drill bit is heavier and prone to deflecting the screw. Furthermore, adjusting the direction when the hollow screw penetrates the two layers of the cortex during placement is difficult with the electric drill. Therefore, the screw along the guide wire direction was first gently tapped, and the direction was confirmed again using fluoroscopy. If the direction was offset, adjustments were made. The screw was subsequently placed if the direction was confirmed by fluoroscopy to be satisfactory. A postoperative CT examination was performed to confirm that the sacroiliac screw was located in the bone and did not penetrate through the bone to the surrounding anatomical structures. Thereafter, only fluoroscopy of the pelvic inlet was needed. The outlet view, pedicle axial view, and inlet view were used to determine whether the sacroiliac screw penetrated the cortex and spinal canal. In contrast, the outlet view was used to determine whether the sacroiliac screw penetrated the superior and inferior nerve root foramina.

In conclusion, the axial view of the pedicle complements the inlet and outlet images, improves the safety, decreases operative time, reduces intraoperative radiation exposure, the effect of intestinal gas is reduced,prevents decreases in sacroiliac joint screw stability (caused by fracture destruction around the screw path through repeated screw placements secondary to poor initial screw placement) and reduces intraoperative injury.

However, the fluoroscopy technique using axial view imaging of the pedicle has certain limitations. First, for the preoperative treatment of patients, appropriate axial view imaging of the pedicle requires higher intestinal tract criteria to be met, and many patients with trauma do not meet the intestinal criteria required for fluoroscopy. Second, injuries to other parts of the spine frequently coexist in patients with fracture dislocation of the sacroiliac joint fracture, making preoperative positioning and fluoroscopy conduction difficult.Finally, due to the complexity of type III fractures of the sacroiliac joint and individual anatomical differences, fluoroscopic axial view imaging of the pedicle requires high level of surgical anatomical knowledge.

The surgical outcomes of percutaneous screw placement for type III fracture dislocation of the sacroiliac joint using axial view imaging of the pedicle include decreased operative time, reduced intraoperative exposure, fewer intraoperative injuries, and greater safety, demonstrating the clinical utility of this method.

## Data Availability

The datasets generated and/or analysed during the current study are not publicly available because we did not obtain authorization from the patients for disclosure regarding patient privacy. However, datasets are available from the corresponding author on reasonable request.
